# Relationships among Indoor, Outdoor, and Personal Airborne Japanese Cedar Pollen Counts

**DOI:** 10.1371/journal.pone.0131710

**Published:** 2015-06-25

**Authors:** Naomichi Yamamoto, Yuuki Matsuki, Hiromichi Yokoyama, Hideaki Matsuki

**Affiliations:** 1 Department of Environmental Health Sciences, Seoul National University, 1 Gwanak-ro, Gwanak-gu, Seoul, Korea; 2 Tokai University Oiso Hospital, 21–1 Gakkyo, Oiso-machi, Naka-gun, Kanagawa, Japan; 3 Department of Nutrition, Kanagawa University of Human Services, 1-10-1 Heisei-cho, Yokosuka-shi, Kanagawa, Japan; 4 Department of Nursing, Tokai University, 143 Shimokasuya, Isehara-shi, Kanagawa, Japan; Peking University, CHINA

## Abstract

Japanese cedar pollinosis (JCP) is an important illness caused by the inhalation of airborne allergenic cedar pollens, which are dispersed in the early spring throughout the Japanese islands. However, associations between pollen exposures and the prevalence or severity of allergic symptoms are largely unknown, due to a lack of understanding regarding personal pollen exposures in relation to indoor and outdoor concentrations. This study aims to examine the relationships among indoor, outdoor, and personal airborne Japanese cedar pollen counts. We conducted a 4-year monitoring campaign to quantify indoor, outdoor, and personal airborne cedar pollen counts, where the personal passive settling sampler that has been previously validated against a volumetric sampler was used to count airborne pollen grains. A total of 256 sets of indoor, outdoor, and personal samples (768 samples) were collected from 9 subjects. Medians of the seasonally-integrated indoor-to-outdoor, personal-to-outdoor, and personal-to-indoor ratios of airborne pollen counts measured for 9 subjects were 0.08, 0.10, and 1.19, respectively. A greater correlation was observed between the personal and indoor counts (*r* = 0.89) than between the personal and outdoor counts (*r* = 0.71), suggesting a potential inaccuracy in the use of outdoor counts as a basis for estimating personal exposures. The personal pollen counts differed substantially among the human subjects (49% geometric coefficient of variation), in part due to the variability in the indoor counts that have been found as major determinants of the personal pollen counts. The findings of this study highlight the need for pollen monitoring in proximity to human subjects to better understand the relationships between pollen exposures and the prevalence or severity of pollen allergy.

## Introduction

Japanese cedar pollinosis (JCP) is known to be a “national affliction” due to its high prevalence among the population and the economic and social implications caused by the disease [1]. JCP is caused by the inhalation of allergenic *Cryptomeria japonica* (Thunb. ex L.f.) D.Don (Japanese cedar) pollen grains, which are dispersed in the early spring throughout the Japanese islands [2, 3]. In the 1950s to 1970s, cedar trees were extensively afforested throughout the Japanese islands due to a policy initiated by the Japanese government [1]. After a few decades, the trees started to release large quantities of pollens into the atmosphere, to which many individuals within the population became sensitized. According to a survey conducted in 2006–2007, 56% of the population was sensitized to *C*. *japonica* antigens, with a 37% prevalence of JCP symptoms [4]. JCP provokes IgE-mediated type I hypersensitivity and induces rhinitis-like symptoms, causing a decline in a patients’ quality of life and lowering their work productivity [5]. The consequent economic burden caused by the need for medications and prescription drugs is thought to be enormous [1]. Therefore, the accurate monitoring of airborne pollens is crucial from the perspective of allergen avoidance or exposure assessments.

The Durham sampler [6] has been traditionally used to quantify airborne cedar pollens in Japan [7, 8]. This sampler uses gravitational settling as a particle collection mechanism, and is suited for the sampling of airborne cedar pollens because of the large settling velocities owing to their size, i.e., approximately 30 μm in aerodynamic diameter [9]. Airborne particles of this aerodynamic diameter can create 2.7 cm sec^-1^ of terminal settling velocity. However, the size of the Durham sampler prevents its use for indoor and personal air sampling. To overcome this drawback, the personal aeroallergen sampler (PAAS), which is a passive personal sampler for airborne coarse particles, has been developed [10]. The PAAS uses gravitational settling to collect airborne coarse particles [10]. A previous study tested its accuracy for the measurement of airborne pollen concentrations by comparing its performance with the results obtained by the reference pump-driven IOM sampler [9]. Furthermore, the PAAS was used to examine the indoor-outdoor relationships of airborne cedar pollens in a school setting [11]. However, no study has yet been conducted to compare the relationships among indoor, outdoor, and personal concentrations of airborne cedar pollens. It is important to characterize personal pollen exposures since the association between pollen exposures and the prevalence of allergic symptoms remains unclear [12–15], in part due to lack of knowledge regarding personal pollen exposures.

The aim of this study was to characterize the relationships between indoor, outdoor, and personal counts of airborne Japanese cedar pollens. Few studies have quantified personal pollen concentrations [9, 16–20]. We conducted a 4-year sampling campaign to measure the indoor, outdoor, and personal counts of airborne allergenic pollens using the PAAS. Additionally, we compared our results with data obtained by the routine ambient pollen-monitoring program of the Japanese government. An accurate assessment of personal pollen exposures is crucial for improving the diagnosis and therapy of pollen allergies [21]. This study provides important insights into how personal pollen counts are related to indoor and outdoor counts, and the accuracy of routine ambient monitoring for the assessment of personal exposures to allergenic pollens.

## Materials and Methods

### Overview

**Fig 1:**
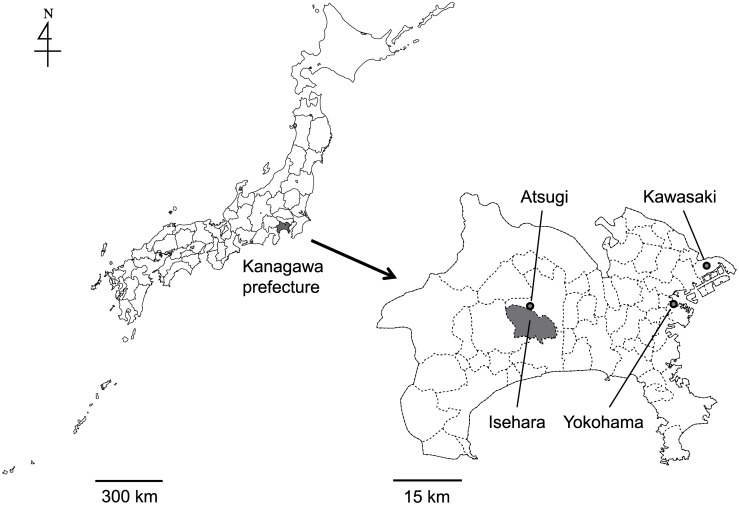
Sampling and monitoring locations. Location of Isehara city, where the indoor, outdoor, and personal pollen sampling was undertaken for study subjects (filled with grey), and the stationary monitoring stations of the Japanese government (circles).

A total of 9 subjects in Isehara city, Kanagawa prefecture, in the suburban Tokyo metropolitan area (Fig 1) were recruited for the measurement of indoor, outdoor, and personal pollen counts in the early spring of 2010–2013. The study area is located in the humid subtropical climate zone, where mean monthly temperatures in Atsugi, a nearby city of Isehara, were 5.3, 8.8, and 13.4°C for February, March, and April, respectively, during the 4-year study period. The site was selected as a bedroom suburb of the Tokyo metropolitan area, and surrounded by the mountainous forests of Japanese cedar trees. Indoor and outdoor airborne pollen counts were measured using the PAAS at representative indoor and outdoor locations in their residences. The subjects were requested to carry the PAAS to measure the personal pollen counts, enabling the relationships among indoor, outdoor, and personal airborne pollen counts to be determined. The outdoor pollen counts measured by the PAAS were further compared with data obtained by the Japanese government from their routine ambient pollen-monitoring program. The three monitoring stations in Kanagawa prefecture, i.e., Atsugi, Kawasaki, and Yokohama (Fig 1), were used to assess the spatial variations in ambient pollen concentrations. The Atsugi station is located near Isehara, whereas the Kawasaki and Yokohama stations are situated about 42 and 30 km east of Isehara, respectively. The research was approved by the ethical committee of Tokai University School of Health Sciences (No. 10–02). Written informed consents were obtained from all participants.

### Pollen sampling and analysis

The PAAS [9–11, 22] (Sibata Scientific Technology, Ltd., Tokyo, Japan) was used for the indoor, outdoor, and personal airborne pollen measurements. A total of 256 sets of indoor, outdoor, and personal pollen samples (768 samples) were collected from a total of 9 subjects over the 4-year monitoring campaign. A mixed cellulose ester membrane filter (25-mm diameter, 0.45-μm pore size; Toyo Roshi Kaisha, Ltd, Tokyo, Japan) immersed in mineral oil was loaded into the sampler as a collection substrate. Airborne pollens were gravitationally collected on the filter substrate, partly covered by a supporting Teflon ring, with an effective sampling area of 3.8 cm^2^.

Five subjects (A, B, C, D, and E) in Isehara participated in 2010, and two different subjects (F and G) participated in 2011. A further two subjects (H and I) participated in 2012–2013. Each subject was requested to conduct simultaneous indoor, outdoor, and personal samplings. Each sampling period lasted for 1–14 days depending on the schedules of the subjects and field technicians, mainly based on availability of human subjects at home and availability of field technicians to visit subjects’ homes. Sampling was repeated consecutively throughout the pollen season, from February 1 to April 30 each year. Each sampling started and terminated on the same date for all subjects. Subjects were recruited based on their availability during the time of our visits to their houses to replace the old samplers with the new ones. All subjects were female.

For the personal sampling, the subjects were requested to carry the PAAS around their neck, when possible. During sleep, the sampler was placed in the participant’s bedroom. The indoor sampling was conducted in a representative indoor location where the subjects spent most of their time, typically in a living room. The outdoor sampling was conducted outside the residential buildings, typically on a balcony or in a yard. Windy locations were avoided to minimize a bias caused by wind as aerosol sampling can be influenced by surrounding wind conditions [9–11, 23]. The indoor and outdoor samplings were terminated around 9:00–9:30, and re-started around 10:00–10:30 using new samplers, containing new collection substrates. Similarly, the personal sampling was terminated around 11:00–11:30 and re-started around 12:00–12:30.

The sampled filter substrates were analyzed in accordance with methods reported elsewhere [9]. Briefly, all pollens collected on the substrate were counted by optical microscopy at ×100 magnification. The entire area of the collection substrate (3.8 cm^2^) was subject to microscopic observation. *Chamaecyparis obtusa* (Siebold & Zucc.) Endl (Japanese cypress) pollens were counted along with Japanese cedar pollens as they are known to be cross-reactive [24]. The airborne pollen quantities measured by the PAAS were reported in units of pollens cm^-2^ day^-1^ or pollens cm^-2^ season^-1^ as reported in numerous epidemiological studies on JCP [2, 3, 12, 13, 25, 26].

### Stationary pollen monitoring data

The ambient pollen concentrations obtained by the routine ambient pollen monitoring undertaken by the Japanese Ministry of Environment were retrieved at http://kafun.taiki.go.jp/library.html (in Japanese). The outdoor pollen concentrations are measured using a fluorescence-based instrument (KH-3000; Yamato Corporation, Kanagawa, Japan) [27] at various sites throughout the Japanese islands, with data made available on an hourly basis. The 2010–2013 data from the three monitoring sites, i.e., Yokohama, Kawasaki, and Atsugi in Kanagawa prefecture (Fig 1), were used in the analyses reported here.

### Data processing and statistical analyses

To compare the hourly pollen data from the Japanese government with the pollen counts measured by the PAAS, the hourly pollen data were averaged over the corresponding measurement periods (i.e., 1–17 days). The pollen counts measured for each sampling period by the PAAS were averaged for all subjects in Isehara. Linear regression was performed to compare the two different methodologies.

Mixed models were used to assess the inter-city and inter-subject variability in the measured airborne pollen concentrations and counts. Pollen concentrations and counts were log-transformed before mixed models. To assess the inter-city variability in the ambient pollen concentrations measured at the three monitoring stations of the Japanese government, the monitoring stations were selected as random effects, whereas the measurement periods were chosen as fixed effect. To assess the inter-subject variability in the indoor, outdoor, or personal counts, the human subjects were chosen as random effects, whereas the sampling periods were selected as fixed effects. The interclass variances (*σ*_α_^2^) were obtained as the intercept variances by the mixed models based the natural-log-transformed pollen concentrations or counts, and the geometric coefficients of variation (CV) were calculated according to the following equation [28]:

$$\text{Geometric CV}=\sqrt{\text{exp}\left(\sigma_{\alpha }^{2}\right)-1}$$ (1)

The geometric CV was used as a measure of the inter-city or the inter-subject variability.

The indoor-to-outdoor (I/O), personal-to-indoor (P/I), and personal-to-outdoor (P/O) relationships were analyzed by mixed models. Owing to the facts that Japanese cedar pollens are exclusively of outdoor origin and indoor cedar pollens originate from outdoor sources, and that the personal exposures occur both indoors and outdoors, we assumed that the indoor counts were dependent on the outdoor counts, and the personal counts were dependent on the indoor and outdoor counts. For each dependent variable, the inter-subject variances (*σ*_α_^2^) as well as the intra-subject variances (*σ*_ε_^2^) were calculated by assuming human subjects as random effects. To assess the relative importance of random inter-subject effects in determining each dependent pollen count (i.e., indoor or personal counts), the intraclass correlation coefficients (ICC) were calculated according to the following equation:

$$\text{ICC}=\sigma_{\alpha }^{2}/\left(\sigma_{\alpha }^{2}+\sigma_{\varepsilon }^{2}\right)$$ (2)

When the indoor counts are selected as a dependent variable and the outdoor counts as an independent variable, the ICC value can represent the relative importance of random inter-subject effects caused by between-subject variability in building-related factors. The building-related factors include air tightness of building and frequency of opening windows/doors. The factors also include type of flooring materials and frequency of floor cleaning. The former category affects residential ventilations, whereas the latter affects indoor pollen emissions in buildings, and they all influence the between-subject variability in the indoor counts in relation to the outdoor counts. When the personal counts are selected as a dependent variable and the indoor and outdoor counts as independent variables, the ICC value can represent the relative importance of random inter-subject effects caused by between-subject variability in non-building-related factors. The non-building-related factors include individual’s activity patterns such as time spent indoors and outdoors.

Additionally, the I/O, P/I, and P/O ratios were calculated with the seasonally-integrated indoor, outdoor, and personal counts for each subject. SPSS version 20 (IBM, Armonk, NY, USA) was used for all statistical tests.

## Results

### Comparison between the PAAS and KH-3000

**Fig 2:**
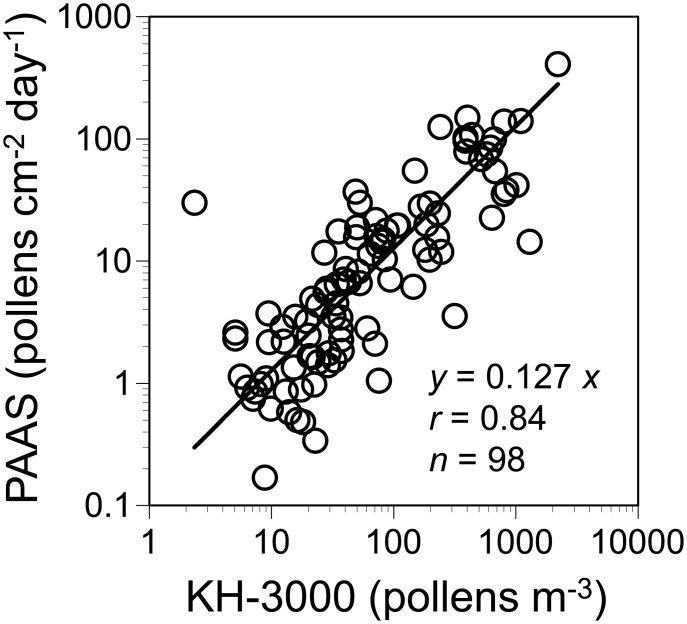
Relationship between the PAAS and KH-3000. The outdoor pollen counts by the PAAS in Isehara are compared with the ambient concentrations by the KH-3000 at the Atsugi station. The pollen counts by the PAAS are averaged for all subjects. The hourly pollen concentrations by the KH-3000 are averaged for the corresponding sampling durations by the PAAS. Linear regression through the origin is used as the intercept is not significantly different from 0.

Fig 2 shows the relationship between the outdoor pollen counts measured by the PAAS in Isehara and the ambient concentrations measured by the KH-3000 real-time pollen monitor at the Atsugi station of the Japanese government. A strong correlation was found between the two methods (*r* = 0.84, linear regression through the origin), suggesting that PAAS is comparable with the KH-3000 to quantify airborne pollen counts with sufficient accuracy.

### Airborne pollen counts

**Fig 3:**
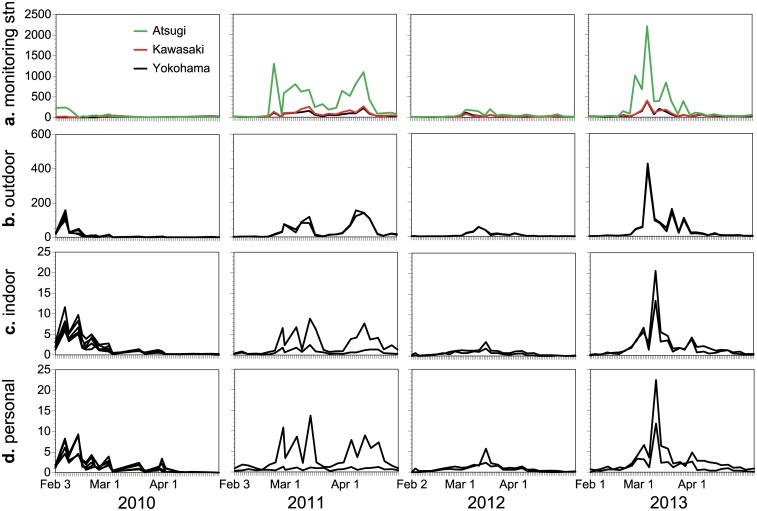
Airborne pollen concentrations and counts during the study periods. Ambient pollen concentrations (pollens m^**-3**^) were monitored at the three stationary monitoring stations of the Japanese government (a). Outdoor (b), indoor (c), and personal (d) pollen counts (pollens cm^**-2**^ day^**-1**^) were measured for each subject in Isehara. Each black line represents data for each subject.

A total of 768 indoor, outdoor, and personal pollen samples were collected from 9 subjects over the 4-year monitoring campaign. Fig 3 shows the time-course of the ambient concentrations, and indoor, outdoor, and personal counts of airborne cedar pollens observed during the 4-year study period. The time-course tendencies in the pollen concentrations and counts differed in each year. At the Atsugi station, the highest outdoor concentration in 2010 was observed on February 8, whereas the highest concentrations in 2012 and 2013 were found in March (Fig 3a). In 2011, two distinct peaks were observed on February 24 and April 11. Similar tendencies were observed for the indoor, outdoor, and personal counts measured by the PAAS among the study subjects (Fig 3b–3d). We also observed the days with the peak indoor and personal pollen counts were preceded by the days with the peak outdoor pollen counts. This tendency was distinct in 2013, with the peak outdoor count observed on March 7 followed by the peak indoor and personal counts on March 11.

### Variability in airborne pollen counts

Spatial variations were observed in the ambient pollen concentrations among the three monitoring stations (Fig 3a). The highest concentrations were found at the Atsugi station with seasonal mean concentrations of 40, 280, 46, and 220 pollens m^-3^ in 2010, 2011, 2012, and 2013, respectively. The corresponding concentrations at Kawasaki were 18, 67, 26, and 57 pollens m^-3^, and at Yokohama were 15, 48, 25, and 47 pollens m^-3^, i.e., the 4-year average concentrations monitored at Kawasaki and Yokohama were 29% and 23% of those monitored at Isehara, respectively. The inter-city variation in the ambient pollen concentrations among the three monitoring sites was 64% in terms of geometric CV (Table 1), suggesting substantial inter-city variations in the ambient concentrations. The intra-city variation was smaller, with a geometric CV value being 11% of those for the outdoor counts measured by the PAAS among the subjects in Isehara (Table 1). The inter-subject variations in the indoor and personal counts were greater than that in the outdoor counts. The geometric CV values for the indoor and personal counts among the different human subjects in Isehara were 43% and 49%, respectively (Table 1).

**Table 1:** Inter-city and inter-subject variability in the airborne pollen concentrations or counts due to random interclass effects.

Type	Number of classes ^a^	Interclass variance (*σ*_α_^2^) ^b^	Geometric CV (%) ^c^
Monitoring station	3	0.34	64
Indoor	9	0.17	43
Outdoor	9	0.013	11
Personal	9	0.21	49

^a^ Number of monitoring stations or human subjects.

^b^ Intercept variances were calculated by the mixed models assuming the monitoring stations or the human subjects as random effects and the measurement days as fixed effects. The natural-log-transformed pollen concentrations (pollens m^-3^) or counts (pollens cm^-2^ day^-1^) were used for the mixed models.

^c^ Geometric coefficient of variation (CV) was calculated according to eq (1).

### Relationships among ambient concentrations, and indoor, outdoor, and personal pollen counts

**Fig 4:**
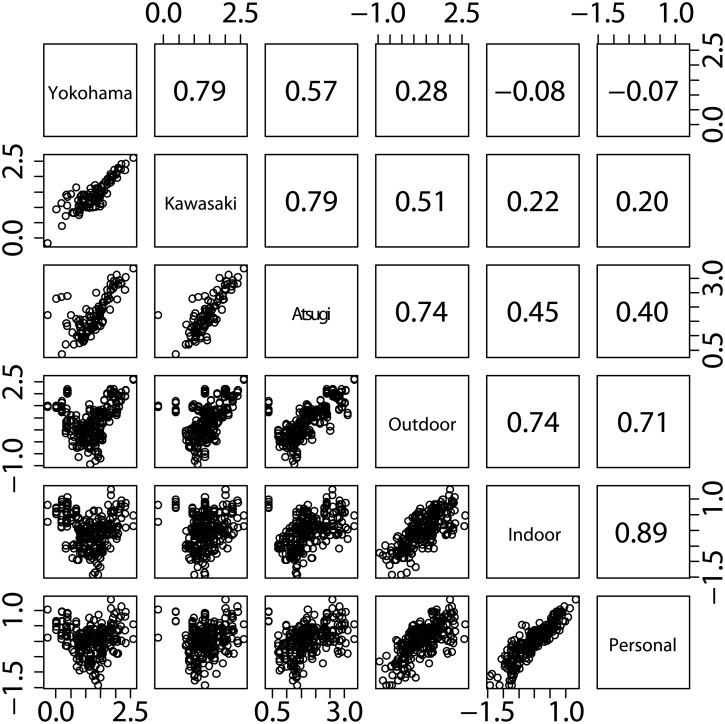
Relationships among the ambient concentrations, and indoor, outdoor, and personal airborne pollen counts. Ambient pollen concentrations monitored at the Yokohama, Kawasaki, and Atsugi stations are reported in the base 10 logarithm of pollens m^**-3**^. Outdoor, indoor, and personal pollen counts measured for each subject in Isehara are reported in the base 10 logarithm of pollens cm^**-2**^ day^**-1**^. Each data point represents measurements for each sampling period. Measurements for all subjects are included for the outdoor, indoor, and personal pollen count data. The values in the upper panels indicate the Pearson product-moment correlation coefficients based on the log-transformed pollen concentrations or counts.

Fig 4 shows the relationships among the ambient concentrations, and indoor, outdoor, and personal counts of airborne cedar pollens. Among the indoor, outdoor, and personal counts, the strongest correlation was found between the personal and indoor counts (*r* = 0.89), whereas the weakest correlation was between the personal and outdoor counts (*r* = 0.71). No or weak correlations were found between the personal counts measured for the subjects in Isehara and the ambient concentrations measured at the stationary monitoring sites, i.e., *r* = 0.40, 0.20, and -0.07 at the Atsugi, Kawasaki, and Yokohama stations, respectively (Fig 4). The mixed model showed an ICC value of 34% for the indoor counts by selecting the outdoor counts as an independent variable (Table 2). An ICC value of 21% was observed for the personal counts by selecting the indoor and outdoor counts as independent variables (Table 2). The I/O, P/O, and P/I ratios were calculated based on the seasonally-integrated indoor, outdoor, and personal counts for each study subject (Table 3). The seasonally-integrated I/O, P/O, and P/I ranged from 0.02–0.19, 0.02–0.19, and 0.59–1.44, respectively, with the corresponding median values of 0.08, 0.10, and 1.19.

**Table 2:** Mix model analyses of inter- and intra-subject variances of each dependent airborne pollen count ^a^.

Dependent	Independent	*t*-value	Inter-subject variance (*σ*_α_^2^)	Intra-subject variance (*σ*_ε_^2^)	ICC (%) ^b^
Indoor	Outdoor	21.77	0.25	0.49	34
Personal	Outdoor	1.70	0.07	0.27	21
	Indoor	17.67			

^a^ The natural-log-transformed pollen counts (pollens cm^-2^ day^-1^) were used in the mixed models. The selected independent variables were chosen as fixed effects.

^b^ Intraclass correlation coefficients (ICC) were calculated according to eq (2).

**Table 3:** Summary statistics of the seasonally-integrated indoor, outdoor, and personal airborne pollen counts measured for each subject.

		Counts (pollens cm^-2^ season^-1^)			Ratios		
Year	Subject	Outdoor	Indoor	Personal	I/O	P/O	P/I
2010	A	750	83	118	0.11	0.16	1.43
	B	645	95	116	0.15	0.18	1.22
	C	674	116	129	0.17	0.19	1.12
	D	857	126	82	0.15	0.10	0.65
	E	895	167	99	0.19	0.11	0.59
2011	F	3500	227	302	0.06	0.09	1.33
	G	3241	62	58	0.02	0.02	0.93
2012	H	785	40	57	0.05	0.07	1.44
	I	890	73	95	0.08	0.11	1.31
2013	H	4002	175	143	0.04	0.04	0.81
	I	4421	239	284	0.05	0.06	1.19

Abbreviations: I/O, indoor-to-outdoor ratio; P/O, personal-to-outdoor ratio; P/I, personal-to-indoor ratio.

## Discussion

We used the PAAS, a simple passive sampler, to conduct the 4-year sampling campaign to examine the relationships between indoor, outdoor and personal counts of airborne cedar pollen. Several types of personal sampler are available to measure airborne pollens [9, 10, 16, 17, 21, 29, 30]. Most of them use a portable pump, with which airborne pollens are actively collected by drawing in air (active samplers) [16, 17, 21, 29]. Such volumetric samplers may be generally preferred owing to the simplicity of conversion from collected amounts to concentrations in air [31]. However, a major drawback of active samplers for personal sampling is the noise generated by pumps. This is particularly problematic when subjects are sleeping or when quiet conditions are required. Another difficulty of active samplers is their need for a power source, resulting in practical difficulties that are impossible to resolve in many situations. The use of the PAAS enabled us to perform a total of 768 indoor, outdoor, and personal pollen samplings from a total of 9 subjects over the 4-year monitoring campaign, which provided detailed information of how personal pollen exposures can be related to indoor and outdoor counts.

Gravitational sampling is possible for airborne cedar pollens owing to their size, i.e., approximately 30 μm in aerodynamic diameter [9]. Airborne particles of this aerodynamic diameter can produce 2.7 cm sec^-1^ of terminal settling velocity, as compared to 12 cm sec^-1^ of a filter face velocity created by the IOM sampler, a commonly-used pump-driven personal inhalable sampler [10]. The collection efficiency by the PAAS is further increased by inertial effects under atmospheric turbulence [10], resulting in approximately 30% collection efficiency relative to the IOM sampler when personal samplings performed [9]. In this study, we reconfirmed the PAAS was useful to quantify airborne cedar pollens with a strong correlation being observed between the PAAS and the KH-3000 pollen monitor (Fig 2). The use of the PAAS can also ensure consistency with the settled pollen data traditionally used in numerous epidemiological studies of JCP [2, 3, 12, 13, 25, 26].

Conversion from the settled pollen counts (pollens cm^-2^ day^-1^) to the volumetric concentrations (pollens m^-3^) is possible using the information of a presumed deposition velocity of airborne pollens, sampling duration, sampling surface area, and collected number of pollens, as detailed elsewhere [9, 10]. However, there are factors that can influence this conversion, which include surrounding wind conditions [10] and, in in case of personal air samplings, human thermal plume [32]. As is the case with all aerosol samplers, including active samplers [23], settling methods are influenced by surrounding wind conditions [9–11]. This bias is unavoidable for sampling inertia-dominant giant particles such as pollens [23]. For instance, varied aspiration efficiencies by wind velocity were reported even for the pump-driven IOM sampler, i.e., 75% at 0.5 m sec^-1^ and 20% at 4.0 m sec^-1^ for particles with 30 μm aerodynamic diameter [33]. In this study, although there were biases associated with wind conditions expected differently across types of air samplings (indoor, outdoor, or personal), across sampled locations, and/or across sampling days, we used the settled pollen counts (pollens cm^-2^ day^-1^) as a basis to examine the relationships between indoor, outdoor and personal airborne pollen concentrations, since the unit is traditionally used in numerous JCP studies [2, 3, 12, 13, 25, 26].

Using the PAAS, the distinct temporal tendencies were observed (Fig 3). First, the two distinct outdoor peaks were observed on February 24 and April 11 in 2011 (Fig 3b), where the second peak was thought to be due to cypress pollens [27]. Similar tendencies were confirmed for the indoor and personal counts measured among the study subjects (Fig 3c and 3d). Second, we found the days with the peak indoor and personal pollen counts were preceded by the days with the peak outdoor pollen counts. This was consistent with previous observations [9, 11]. As cedar and cypress pollens are exclusively of outdoor origin, the observed lag times were likely due to the time required for the airborne pollens to infiltrate into indoor environments [34] and then accumulate, or to be brought indoors by the human subjects via attachment to their clothing [35], as well as the release of previously deposited pollen. The observed lag times may implicate potential inaccuracy of use of the outdoor pollen counts as a basis of personal exposures.

There is an increasing number of studies that have reported I/O ratios for airborne pollens [11, 34, 36–39]. However, little is known about the relationship among indoor, outdoor, and personal pollen concentrations such as P/O and P/I. We quantified the I/O, P/O and P/I ratios based on the seasonally-integrated indoor, outdoor, and personal pollen counts. The median I/O, P/O, and P/I ratios were 0.08, 0.10, and 1.2 respectively (Table 3). The residential I/O ratios found in this study appeared to be larger than those previously characterized by the PAAS in a classroom, ranging from 0.04 to 0.06 [11]. A study reported the I/O ratios of *Betula* pollen grains ranging from 0.01 to 0.22, which were characterized by pump-driven active samplers [37]. Though types of pollens analyzed are unknown, a similar I/O (= 0.025) was reported by researchers using button personal inhalable aerosol samplers [36]. Our findings reconfirmed outdoor counts were substantially higher than indoor counts for pollens of exclusively outdoor origin.

We observed the substantial inter-city variability in the ambient pollen concentrations (Fig 3a and Table 1), indicating the importance of the proximity of the stationary monitoring site to the location of potentially affected populations when using outdoor concentrations to predict their exposures. Indeed, no or weak correlations were found between the personal and ambient concentrations measured at the distant sites, i.e., *r* = -0.07 and 0.20 at the Yokohama and Kawasaki stations, respectively, whereas a moderate correlation (*r* = 0.40) was found at the nearest Atsugi station (Fig 4). Meanwhile, the intra-city variability in the outdoor counts was smaller (11% geometric CV) than the inter-city variability in the ambient concentrations (64% geometric CV) (Table 1), indicating distance as an important factor in determining outdoor concentrations.

Personal pollen exposures can be influenced not only by outdoor concentrations but also by indoor concentrations. Since people spend most of their time indoors [40–42], indoor concentrations are likely major determinants of personal pollen exposures. Indeed, the larger *t*-value was observed for the indoor counts than for the outdoor counts by the mixed model (Table 2). The stronger correlation was also found between the personal and indoor counts (*r* = 0.89), than between the personal and outdoor counts (*r* = 0.71) (Fig 4). Owing to its significance, it is important to characterize how indoor pollen counts vary across each residence.

We observed the greater inter-subject variability in the indoor counts (43% geometric CV) than in the outdoor counts (11% geometric CV) for the subjects living in the same city (Table 1 and Fig 3). The observed variation was partly due to between-subject variability in air tightness of residential building, frequency of opening windows/doors, type of flooring materials, frequency of floor cleaning, and so on. Our mixed model analysis showed 34% of variability in the indoor counts was due to such random inter-subject effects associated with the between-subject variability in building-related factors (Table 2). Since the indoor counts are the most important determinant of the personal pollen counts, the variability in the indoor counts likely propagates the variability in the personal counts. Additionally, the between-subject variability in the non-building-related factors (e.g., time spent indoors and outdoors) could also affect the personal pollen counts, with 21% of variability in the personal counts attributable to such random inter-subject effects (Table 2). These random inter-subject effects are thought to propagate between-subject variations in the personal counts. Indeed, the largest inter-subject variability was observed in the personal counts (49% geometric CV) (Table 1), highlighting the potential need for personal pollen sampling to provide more accurate exposure assessments.

To date, most epidemiological studies of JCP have relied on ambient pollen counts as a basis to estimate personal pollen exposures [12–15]. However, the association between pollen exposures and the prevalence of allergic symptoms remains unclear [12–15], in part due to lack of knowledge regarding personal pollen exposures. In the last decade, substantial efforts have been made by the Japanese government to establish an ambient pollen-monitoring network to determine the outdoor concentrations on a real-time basis [25, 27]. Such a system should be complementary to more detailed personal monitoring when used in association with more detailed personal monitoring, as our results indicate a potential inaccuracy in the use of ambient concentrations as a basis for estimating personal exposures. Our findings indicate personal pollen exposures can be influenced more greatly by indoor counts than by outdoor counts. We also observed the large between-subject variations in the indoor and personal pollen counts due to random inter-subject effects. The findings of this study emphasize the potential need for personal pollen monitoring, for instance by using the PAAS, in future epidemiological studies of JCP to better understand the relationships between pollen exposures and the prevalence or severity of pollen allergy symptoms.
